# Supplementary material for: Identification of photoperiod-induced specific miRNAs in the adrenal glands of Sunite sheep (Ovis aries)
Source: Front Vet Sci. 2022 Jul 22;9:888207. doi: 10.3389/fvets.2022.888207 (PMC9354845; doi:10.3389/fvets.2022.888207)
Supplement: Supplementary file 1 [file Data_Sheet_1.ZIP › Supplementary file/miRDeep2 file.zip!/miRDeep2 file/Know_Structure/LP42A1/oar-mir-379.pdf]

miRBase precursor : oar-mir-379
Total read count : 32187
oar-miR-379-5p read count : 30555
oar-miR-379-3p read count : 1621
remaining reads : 11

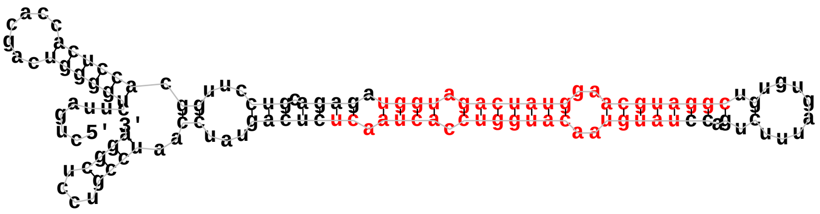

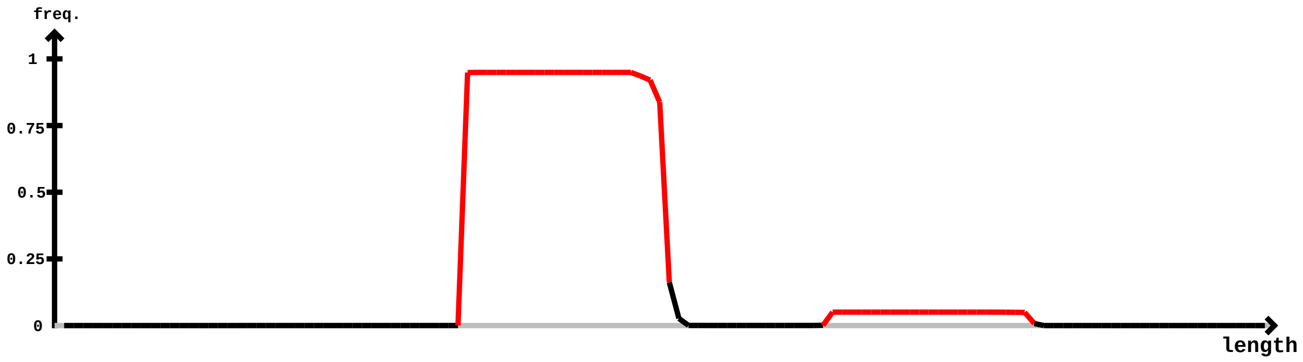


```
                                         oar-miR-379-5p                       oar-miR-379-3p
5'- cugauucuggggucagcaccacuccacgguuccugcagagaugguagacuauggaacguaggcugugugauuucugaccuauguaacaugguccacuaacucucaguauccaauccguccucgga -3'   exp
    .......((((((.......)))))).((...(((.((((.((((.(((((((..((((((((.(........).).)))))))..))))))).)))).)))))))...))..((((....))))   reads   mm
    ................caccacuccacgguuccugcagaga....................................................................................   1       0
    ..................ccacuccacgguuccugcagaga....................................................................................   1       0
    ........................................augguagacuauggaacguag................................................................   2       0
    ........................................augguagacuauggaacguagg...............................................................   4       0
    .........................................ugguagacuauggaacg...................................................................   2       0
    .........................................ugguagacuauggaacgu..................................................................   430     0
    .........................................ugguagacuauggaacgua.................................................................   498     0
    .........................................ugguagacuauggaacguag................................................................   2660    0
    .........................................ugguagacuauggaacguagg...............................................................   21736   0
    .........................................ugguagacuauggaacguaggc..............................................................   4359    0
    .........................................ugguagacuauggaacguaggcu.............................................................   836     0
    .........................................ugguagacuauggaacguaggcug............................................................   3       0
    .........................................ugguagacuauggaacguaggcugu...........................................................   3       0
    .........................................ugguagacuauggaacguaggcugugu.........................................................   1       0
    .........................................ugguagacuauggaacguaggcugugugauuucugac...............................................   1       0
    ..........................................gguagacuauggaacguagg...............................................................   15      0
    ..........................................gguagacuauggaacguaggc..............................................................   6       0
    ..............................................................cugugugauuucugacc..............................................   6       0
    ..............................................................cugugugauuucugacU..............................................   2       1
    ..............................................................................cuauguaacaugguccacuaac.........................   23      0
    ..............................................................................cuauguaacaugguccacuaacu........................   2       0
    ...............................................................................uauguaacaugguccacu............................   4       0
    ...............................................................................uauguaacaugguccacA............................   1       1
    ...............................................................................uauguaacaugguccacC............................   3       1
    ...............................................................................uauguaacaugguccacua...........................   12      0
    ...............................................................................uauguaacaugguccacuaG...........................   2       1
    ...............................................................................uauguaacaugguccacuaa..........................   13      0
    ...............................................................................uauguaacaugguccacuaac.........................   1312    0
    ...............................................................................uauguaacaugguccacuaacu........................   245     0
    ...............................................................................uauguaacaugguccacuaacuc.......................   1       0
    ................................................................................auguaacaugguccacuaac.........................   2       0
    ................................................................................auguaacaugguccacuaacu........................   1       0
```
